# Direct binding of MEK1 and MEK2 to AKT induces Foxo1 phosphorylation, cellular migration and metastasis

**DOI:** 10.1038/srep43078

**Published:** 2017-02-22

**Authors:** Shiri Procaccia, Merav Ordan, Izel Cohen, Sarit Bendetz-Nezer, Rony Seger

**Affiliations:** 1 Department of Biological Regulation, The Weizmann Institute of Science, Rehovot, Israel

## Abstract

Crosstalk between the ERK cascade and other signaling pathways is one of the means by which it acquires its signaling specificity. Here we identified a direct interaction of both MEK1 and MEK2 with AKT. The interaction is mediated by the proline rich domain of MEK1/2 and regulated by phosphorylation of Ser298 in MEK1, or Ser306 in MEK2, which we identified here as a novel regulatory site. We further developed a blocking peptide, which inhibits the interaction between MEK and AKT, and when applied to cells, affects migration and adhesion, but not proliferation. The specific mechanism of action of the MEK-AKT complex involves phosphorylation of the migration-related transcription factor FoxO1. Importantly, prevention of the interaction results in a decreased metastasis formation in a breast cancer mouse model. Thus, the identified interaction both sheds light on how signaling specificity is determined, and represents a possible new therapeutic target for metastatic cancer.

The mammalian cell is under constant stimulation from its surroundings and needs to respond accurately and rapidly in order to function properly and survive. Most extracellular ligands stimulate cellular processes via intracellular signal transduction pathways, which are the main routes of communication between the plasma membrane and intracellular compartments^1^. Two major signaling pathways that operate in a large number of cells and conditions are the Extracellular signal-Regulated Kinase 1 and 2 (ERK1/2)^2^, and the phosphatidylinositol 3-kinase (PI3K)/AKT pathways^3^. Each of them induces different and even conflicting cellular reactions including proliferation, differentiation, survival and migration. Several molecular mechansims governing the specificity of each pathway include: duration and strength of the signals, scaffolding, localization and crosstalk between distinct cascades^4^. However, this important signaling issue is still far from being resolved.

Upon growth factor stimulation, the ERK1/2 cascade transmits its signals via a sequential phosphorylation and activation of a core three tires of protein kinases, Raf, MEK1/2, and ERK1/2 downstream of Ras^5^,^6^. Within the cascade, the MAPKK components, MEK1 and MEK2, are both 45 kDa dual specifcity kinases that are activated by their upstream activators via phosphorylation of two adjacent Ser residues within their activation loop (Ser 218 and 222 in MEK1^7^,^8^,^9^). Once activated, they phosphorylate both Tyr and Thr residues of ERK1/2 to induce the activation of the latter. Notably, ERK1/2 are the sole substrates to be efficiently activated by the MEK1/2, suggesting that the latter serve as specificity determining components of the ERK cascade. While MEK1/2 are specific to ERK1/2 in phosphorylation, they can act out of the ERK1/2 cascade by protein-protein interactions^10^,^11^. There are three main interacting domains in MEK1. MEK1’s N-terminus which contains a basic D domain interacts mainly with ERK1/2 to allow the subsequnt phosphorylation, but was shown to interact with PPARγ^10^ and Grb10^12^ as well. Another site is the Domain of Variable Docking (DVD) that interacts with the upstream activators of MEK1/2^13^. Finally, MEK’s Proline Rich Domain (PRD) was shown to be involved in binding to MP-1^14^, CDK5^15^, MyoD^16^ and to MAGI1 as part of a complex with PTEN^17^. Little is known regarding MEK2 protein-protein interactions, but it was shown to heterodimerize with the MEK1 isoform^18^.

The PI3K/AKT pathway is another major intracellular signaling pathway that acts as a critical signaling node in diverse cellular responses^19^. AKT, which is also known as Protein Kinase B (PKB) is a Ser/Thr kinase. In mammals there are three 60 kDa isoforms of AKT encoded by different genes, which share a similar structure of three domains: A Pleckstrin Homology (PH) domain in the N-terminus, a C-terminus necessary for induction and maintenance of AKT’s kinase activity (the Regulatory domain) and between those a hinge region of a kinase domain with Ser/Thr specificity^20^,^21^. The structural homology between the AKT isoforms extends to their activation mechanism. The activity of all AKTs is induced following activation of the lipid kinase PI3K, which, upon stimulation, forms D3-phosphorylated lipids (PIP_3_). The formed phospholipids then recruit the PH domain of AKT to their vicinity, and this anchors the kinase to the plasma membrane. There, the AKT undergoes two phosphorylations, on Thr308 by PDK1 and on Ser473 mainly by mTORC2 that cause full activation of the kinase. The activity of the pathway is negatively regulated by the lipid phosphatase PTEN^22^, and by direct dephosphorylation of AKT by PP2A and PHLPP^21^,^23^. Activated AKT affects the cell via phosphorylation of numerous substrates including Glycogen Synthase Kinase 3 (GSK-3^24^); TSC2 and PRAS40 that lead to the activation of mTORC1^25^; Inhibitor of Nuclear Factor Kappa B (IKK1) that affects the NFkB signaling^26^; mdm2 that affects p53^27^; The transcription factor Forkhead Box protein O (FOXO^28^) and many more. Dysregulation of AKT signaling including its downstream effectors is inviolved in cancer and other pathologies^29^.

Previous studies have shown the existence of intricate interplay between several components of the ERK1/2 cascade and the PI3K/AKT pathway, including cross-inhibition, cross-activation and pathways convergence^30^. The most prominent cross talk is seen in the upstream level as Ras was shown to activate the PI3K complex^31^ via a direct interaction with its p110 catalytic subunit^32^. This interaction can also positively affect the ERK1/2 cascade^33^,^34^. Another component of the PI3K pathway that interacts with the ERK cascade is PTEN, which forms a complex with MEK1 that is important for its membrane recruitment^17^. Cross-inhibition was shown most notably in the case of Raf phosphorylation by AKT. In response to various stimulations, AKT can directly phosphorylate Ser259 of C-Raf^35^,^36^,^37^, and Ser364 of B-Raf^38^, and this phosphorylation promotes Raf interaction with the inhibitory scaffold 14–3–3. Interestingly, AKT can also activate the ERK1/2 cascade via direct phosphorylation of Ser338 of C-Raf^39^,^40^. Thus, AKT can differentially regulate the ERK1/2 cascade in a time- and dose-dependent manner, depending upon the biological context. Cross-activation between the two pathways was also shown for PAK1, which is activated downstream to PI3K^41^, and then directly phosphorylates MEK1 on Ser298 in response to adhesion to fibronectin, leading to MEK1’s enhanced activation^42^,^43^. There is also evidence that PAK3, another kinase downstream of PI3K, directly phosphorylates Raf-1 on Ser338 and enhances its activity^44^. Pathway convergence can also occur downstream of the two pathways as in some cases, both cascades can affect the same targets, as in the case of the transcription factor FoxO3^45^ or the central translation regulating target mTORC1^46^.

Since both the ERK1/2 and the PI3K/AKT signaling pathways play critical roles in the transmission of cellular signals, components of these pathways are frequently mutated, hyper-activated, or aberrantly expressed in many human cancers^47^,^48^. For this reason, components of both pathways are often targeted in the development of anticancer therapeutic agents^49^,^50^. However, inhibition of the ERK1/2 cascade results in resistance of the cancer cells by compensatory activation of PI3K pathway signaling^51^,^52^,^53^, and vice versa; inhibition of PI3K pathway components confers resistance by increased ERK cascade signaling^54^,^55^. Therefore, combined inhibition therapy is thought to be more effective and synergetic, and ultimately achieve better cancer treatment^56^,^57^,^58^.

Here we identified a novel interaction between AKT of the PI3K/AKT pathway and both MEK1 and MEK2 of the ERK cascade. The interaction is mediated by the PRD of MEK1 and MEK2, and is regulated by phosphorylation of Ser298 in MEK1 or Ser306, which we identified as a phosphorylation site in MEK2. Utilizing a peptide that interferes with the interaction we found that the interaction is required for FOXO1 phosphorylation, cellular migration, and cellular adhesion in tissue culture cells, as well as emergence of metastasis in nude mice. However, it did not significantly affect cellular proliferation or tumor growth in mice. Hence, the MEK-AKT interaction extends the signaling repertoire of the pathways, essentially creating a third independent pathway, and suggests a novel target for treating methastasis formation.

## Results

### A direct interaction of MEK1/2 with AKT1/2/3 is increased upon EGF stimulation

**Figure 1:**
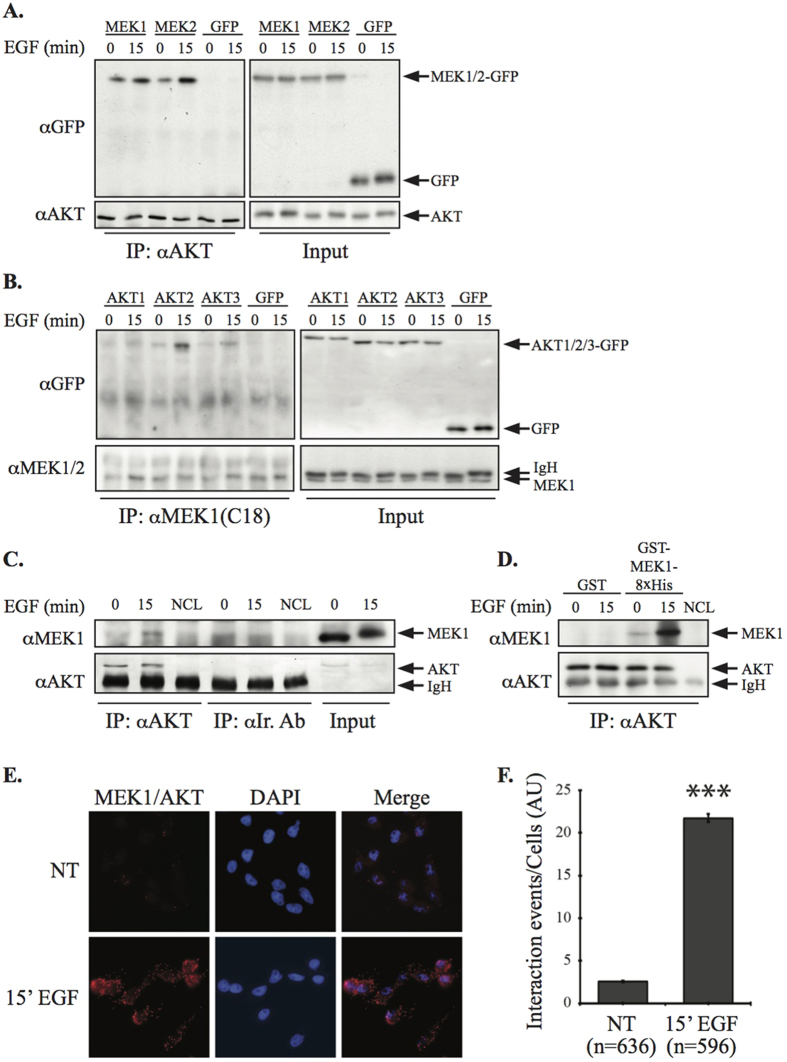
MEK1/2 interact with AKT1/2/3 *in vitro* and *in vivo*. (**A**) CoIP of AKT with overexpressed MEK. COS-7 cells were transiently transfected with MEK1-GFP, MEK2-GFP and GFP control plasmids. When the cells reached sub-confluence they were serum starved (0.1% FBS, 18hrs), and then either stimulated with EGF (50 ng/ml, 15 min) or left untreated. The cytosolic extracts were subjected to CoIP with αAKT Ab. The amount of interacting MEK1-GFP, MEK2-GFP or AKT was determined by Western blotting. (**B**) Reciprocal CoIP of MEK with overexpressed AKT. COS-7 cells were transiently transfected with AKT1-GFP, AKT2-GFP, AKT3-GFP and GFP control plasmids. The cytosolic extracts of either EGF-stimulated or untreated COS-7 cells were subjected to CoIP with αMEK1(C18) Ab. The amount of interacting AKTs and loaded MEK was determined by Western blotting with the indicated Abs. (**C**) CoIP of endogenous MEK1 and AKT. HeLa cells were serum starved at sub-confluence and then either stimulated with EGF or left untreated. The cytosolic extracts were subjected to CoIP with either αAKT or total IgG (irrelevant Abs). The amount of interacting MEK and loaded AKT was determined by western blotting. NCL – No Cell Lysate control. (**D**) *In vitro* interaction of IPed AKTs with recombinant MEK1. AKT was IPed from HeLa cells treated as described in A by sequential washes once with RIPA buffer, then twice with 0.5 M LiCl and twice with Buffer A. Recombinant GST-MEK1–8xHis or GST alone control (500 ng) were incubated with the bound AKT in the presence of 0.1% BSA to avoid non-specific interactions. Interaction and loading was detected by Western blotting. (**E**) Representative PLA of MEK1 and AKT. HeLa cells were grown on cover slips to 70% confluence, serum-starved, and then either stimulated with EGF (50 ng/ml, 15 min), or left untreated. Cells were subjected to a PLA assay using the αMEK1(H8) Ab together with αAKT Ab. The nuclei were detected using DAPI, and slides were visualized using a fluorescent microscope (x40 magnification). (**F**) Quantification of the results in E. Quantification was performed using ImageJ analysis tool for ~200 cells in each experiment. Data shown represents mean ± S.E.M. p < 0.0001 (treated vs. control).

Using anti AKT antibody as a control in a non-related co-immunprecipitation (CoIP) experiment, we found to our surprise that MEK1/2 was present in the precipitate. In order to confirm this initial finding, we overexpressed either MEK1-GFP or MEK2-GFP in COS7 cells and found that both MEKs are indeed able to specifically CoIP with the endogenous AKTs (Fig. 1A). Importantly, the detected interaction increased upon EGF stimulation of the Cos7 cells, and similar results were obtained in HeLa cells as well (Data not shown). The same trend was seen in a reciprocal experiment, where overexpressed AKT1-GFP, AKT2-GFP and AKT3-GFP were CoIPed with endogenous MEK1/2 in COS-7 cells (Fig. 1B). The AKT2 isoform demonstrated notably stronger binding to MEK than the other two isoforms, indicating that this is the main isoform that CoIPs with the endogenous MEKs. In order to verify that the interaction observed also occurs between both endogenous proteins, we performed a CoIP between the endogenous MEK1 and AKTs in HeLa cells (Fig. 1C). Indeed, the endogenous MEK1 interacted with the endogenous AKTs, and their interaction upon stimulation was increased as well. In order to determine if MEK1/2 and AKT interact directly and not via a mediator protein, we performed an *in vitro* binding assay (Fig. 1D). In this assay AKT was Immunoprecipitated (IPed) from HeLa cells, followed by stringent washes that are meant to shed off all residual interacting proteins such as chaperones or scaffold proteins. This way of purification was important since our previous results indicated that AKT may require additional phosphorylations (other than its activatory once) for the binding, that can not be mimicked by expression in bacteria (data not shown). This purified protein readily interacted with a recombinant GST-MEK1-8xHis, but not with a recombinant GST protein control. Finally, we verified the interaction between the endogenous MEK1 and AKTs by Proximity Ligation Assay (PLA). As expected, we found that MEK1 and AKT interacted, and the number of interaction events is significantly increased upon 15′ EGF stimulation (Fig. 1E,F). A recent work suggested MEK1 and MEK2 form a heterodimer^18^, therefore it was important to find out whether the binding of MEK2 to AKT is direct, or mediated by MEK1. Using MEK1^−/−^ mouse embrionic fibroblast (MEFs) we found that MEK2 indeed interacts with AKT, and this was slightly increased upon stimulation (Fig. S1). We attribute the limited increase in interaction upon stimulation in these knockout cells to a reduced EGF effect on AKT activation. Importantly, using the *in vitro* binding assay described for MEK1, we further found that MEK2′s interaction is indeed direct and not mediated by MEK1 or any other adaptor. Finally, we overexpressed the three AKTs in COS7 cells and MEK1^−/−^ MEFs and showed that MEK2 exhibits better binding to AKT3 than to AKT1 and AKT2 both in resting and in stimulated cells (Fig. S1). Thus, these results indicate that although MEK1 and MEK2 can interact with all three AKT isoforms, they have distinct affinities to these isoforms, where MEK1 favors binding to AKT2 and MEK2 favors AKT3.

### Phosphorylation of Ser298 in MEK1 and Ser306 in MEK2 regulates their interaction with AKT

**Figure 2:**
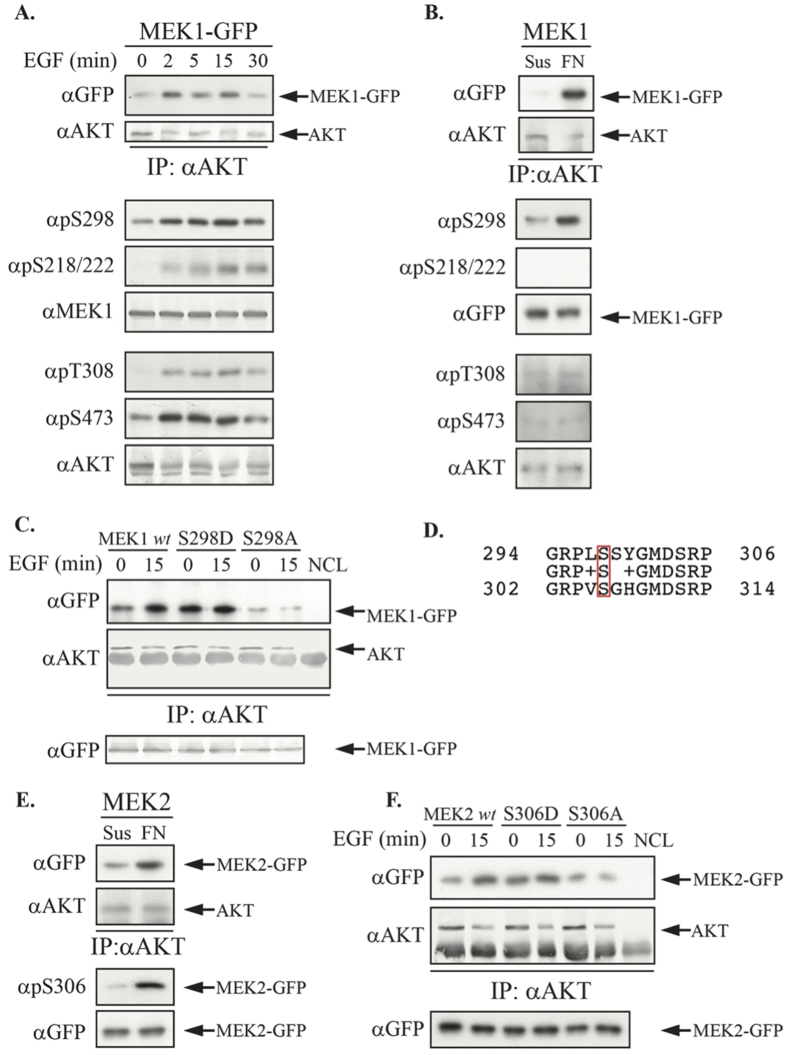
MEK1/2-AKT interaction is regulated by phosphorylation of Ser298 in MEK1 and Ser306 in MEK2. (**A**) Time course interaction and phosphorylation of MEK1 and AKT upon binding. COS-7 cells were transiently transfected with MEK1-GFP. When the cells reached sub-confluence they were serum starved (0.1% FBS, 18 hrs), and then either left untreated or stimulated with EGF (50 ng/ml) for the indicated times. The cytosolic extracts were subjected to CoIP with αAKT Abs. The amount of interacting MEK1-GFP and loaded AKT as well as MEK1 and AKT phosphorylation were determined by Western blotting. (**B**) Interaction and phosphorylation of MEK1 and AKT upon FN binding. COS-7 cells were transfected and starved as in A. The cells were harvested with Trypsin C, followed by its neutralization with SBTI (100 μg/ml), then held in suspension for two hours. Half of the cells were seeded on plates precoated with FN (10 μg/ml) for 30 min and the rest remained in suspension. The cells were harvested and subjected to CoIP and Western blotting as in A. (**C**) Ser298 mutants interact differently with AKT compared to *wt*. COS-7 cells were transiently transfected with MEK1-GFP *wt* or its mutants S298D and S298A. The cells were subjected to CoIP as described in A. The amounts of the overexpressed proteins were determined by Western blotting. (**D**) MEK1-MEK2 Sequence alignment. Amino acid pairwise alignment shows Ser in position 306 of MEK2 corresponding to position 298 in MEK1. (**E**) MEK2’s Ser306 phosphorylation in response to FN. COS-7 cells were transiently transfected with MEK2-GFP and treated as in Fig. 1C. The amount of interacting MEK2-GFP, loaded AKT and MEK2’s pSer306 site was determined by Western blotting. (**F**) Ser306 phosphomimetic and non-phosphorylatable mutants modulate the binding of MEK2 with AKT. COS-7 cells were transiently transfected with MEK2-GFP *wt* or its phosphomimetic and non-phosphorylatable mutants, S306D and S306A respectively. The cells were subjected to CoIP with αAKT Abs. The amount of interacting MEK2-GFP, and AKT was determined by Western blotting Western blots were developed with either NBT/BCIP or ECL.

As the interaction of MEK with AKT was increased upon stimulation, we suspected that stimuli-dependent phosphorylation of one of the known phosphorylation sites might regulate it. To examine this hypothesis we used a time course assay, in which the correlation between the MEK-AKT interaction and the phosphorylation of activatory sites was tested for both kinases (Fig. 2A). Interestingly, we found that the phosphorylation of MEK1’s Ser298 (but not Ser218/22), as well as the activatory phosphorylations of AKT Ser473 and Thr308 was highly correlated to the interaction observed between overexpressed MEK1-GFP and AKT. It was previously shown that Ser298 in MEK1 is phosphorylated by PAK1 upon adhesion to fibronectin (FN^42^,^43^). Therefore, we tested the MEK-AKT interaction in suspension or 30 min after cell adhesion to FN (Fig. 2B). Under these conditions, MEK1 readily interacted with AKT, while only the Ser298 in MEK1, not Ser218/222-MEK1 or Thr308/Ser473-AKT, was significantly phosphorylated. Therefore, Ser298 phosphorylation is the only one correlated with the increased MEK1- AKT binding upon FN adhesion. To further verify the involvement of Ser298 in the regulation of MEK1-AKT interaction we generated phosphomimetic and non-phosphorylatable mutants, S298D and S298A respectively, and tested their interaction with AKT (Fig. 2C). While the interaction of *wt* MEK1 was increased upon stimulation with EGF, the interaction of S298D mutant was constantly high, and the interaction of S298A was even lower than basal, regardless of stimulation.

Finding that MEK2 interacted with AKT similarly to MEK1, we hypothesized that it has a phosphorylation site that can regulate the interaction as well. Such phosphorylation has never been reported, therefore, we undertook to identify and characterize it. Indeed, alignment between the amino acid sequences of MEK1 and MEK2 discovered a Ser306 in MEK2, as a potential corresponding phosphorylation site (Fig. 2D). This site was shown to be phosphorylated in a wide MS effort to identify phosphorylation sites, and is listed on phosphosite. In order to test this candidate residue, we used an antigenic peptide based on the phosphorylated sequence to generated phosphosite-specific αpS306MEK2 Ab (Fig. S2). As expected, the Ab recognized the MEK2-GFP that was IPed from resting COS7 cells, and this recognition was increased upon EGF stimulation. Competition with the antigenic peptide completely abolished the recognition validating the specificity of the Ab. Moreover, the Ab only marginally recognized the S306A mutant but did recognize the phosphomimetic S306D to some extent (Fig. S2B). There was a marginal recognition of MEK1-GFP, but this was not affected by EGF stimulation (data not shown). Finally, the Ab recognized the endogenouse protein as well (Fig. S2C) and this recognition was increased with adhesion to FN. This data indicated that the Ab is indeed specific to pSer306-MEK2. We then further studied the role of the Ser306-MEK2 phosphosphorylation and found that as in the case of MEK1, upon adhesion to FN, MEK2 was phosphorylated, and this increase in phosphorylation on Ser306 was correlated with the increase in MEK2-AKT interaction (Fig. 2E). Next we tested the interaction of MEK2′s S306D and S306A mutants with AKT before and after EGF stimulation (Fig. 2F). Here also, as is the case for MEK1, the interaction of S306D was high both in basal and upon stimulation, and the interaction of S306A was even lower than the basal, regardless of stimulation. Thus, we show for the first time that Ser306-MEK2 is phosphorylated upon stimulation and as for Ser298-MEK1, the phosphorylation is important for the interaction with AKT.

### MEK1 interacts with AKT via its Proline Rich Domain

**Figure 3:**
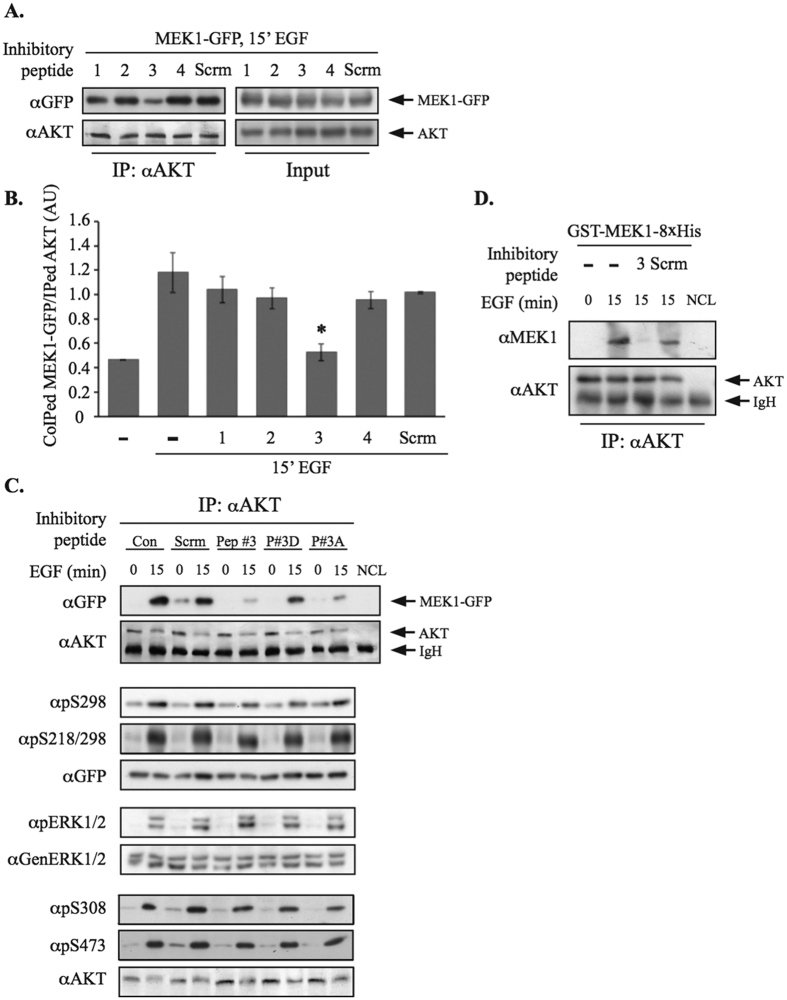
Peptide#3 inhibits MEK1-AKT interaction. (**A)** Peptides scan. COS-7 cells were transiently transfected with MEK1-GFP. Sub-confluent cells were serum starved (0.1% FBS, 16hrs), added the indicated myristoylated peptide for 2 hrs, and then stimulated with EGF for 15 min. The cytosolic extracts were subjected to CoIP with αAKT Abs. The amount of interacting MEK1-GFP was determined by western blotting with αGFP Abs. The same membrane was blotted with αAKT Abs. The equal input is shown in the *right panels*. (**B**) Densitometric analysis of three experiments as shown in A. (**C**) Peptide#3 does not significantly affect MEK/AKT phosphorylations. COS-7 cells were treated as in A with the indicated peptides and subjected to CoIP. The cytosolic extracts were also subjected to Western blotting with the indicated Abs. (**D**) Peptide#3 inhibits MEK1-AKT binding *in vitro*. AKT was IPed from COS-7 cells stimulated as indicated, by sequential washes. Recombinant GST-MEK1–8xHis (500ng) was incubated with the bound AKT in the presence of peptide as stated and of 0.1% BSA. Interaction was detected by western blotting with αMEK1(H8) Abs. The equal amount of IPed AKT was determined by western blotting with αAKT Ab.

Next, we undertook to identify the region in MEK1 responsible for the interaction. Since Ser298 was shown to be important for the interaction, we hypothesized that the binding site lies within the PRD. To examine this possibility and identify the exact binding site, we designed four peptides covering distinct parts of the PRD (Fig. 3A). In order to allow a rapid penetration of the peptides through the cell membrane and layover in the cytoplasm, they were conjugated to myristic acid in their N-terminus as recently reported^59^. As expected, the peptides were found localized in the cytoplasm of the cells 2 hrs after treatment, and were still detectable there 22 hrs later (Fig. S3). We found that the myristoylated Peptide#3, but not the other peptides, successfully inhibited the MEK1-AKT interaction in our system (Fig. 3A,B). Since this peptide includes Ser298 it was possible for this residue to regulate and mediate the interaction by itself. To confirm the importance of this region and to examine the relative role of Ser298, we prepared a MEK1 construct in which Ser298 and 7 adjacent amino acids within the peptide were replaced by Ala, and compared it to the S298A construct. Interestingly, both mutants significantly reduced the MEK1-AKT binding, but the effect of this 8 amino acid mutant was more pronounced than the effect of Ser298A alone (Fig. S4). Therefore, we concluded that the MEK1-AKT binding is mediated mainly by the phosphorylated Ser298, but other adjacent residues in the PRD contribute to this interaction as well.

We then undertook to further characterize the peptide. In a previous study^59^, we found that peptides with modulated phosphorylation site may have a superior effect to peptides based on the WT sequence. To test it for this peptide we designed two additional peptides of the same sequence, in which the position corresponding to Ser298 was changed to either the phosphomimetic Asp (peptide P#3D) or the non-phosphorylatable Ala (peptide P#3A) residues. All three forms of Peptide#3 inhibited the interaction between MEK1-GFP and AKT in a CoIP assay (Fig. 3C, *upper panels*), both in basal and stimulated cells, although the effect of P#3D was smaller than that of the other two. Thus, the WT peptide that demonstrated routinely the most substantial effect was chosen for further characterization. We then undertook to study the specifcity of the peptide. Importantly, none of the peptides significatly affected the phosphorylations of MEK1 (Ser298, Ser218/222) or AKT (Thr308/Ser473). In addition, the peptides did not alter the activatory phosphorylations of either ERK1/2 or AKT’s or their downstream target, GSK3 (Fig. 3C *lower panels*, Fig. S5B). These results confirm the specificity of the peptide to the MEK-AKT interaction, without affecting their activation or other interactions of the kinases. To test whether the peptide directly inhibits the interaction between MEK1 and AKT, and not via a mediator protein, an *in vitro* binding assay was carried out (Fig. 3D). In this experiment, purified AKT was obtained by IP from HeLa cells stimulated with EGF or left untreated followed by extensive washes. This purified protein was added to a recombinant GST-MEK1-8xHis, and their binding was followed by precipitation. Indeed, while the two proteins interact under the conditions used, the addition of Peptide#3, and not the scrambled peptide control, inhibited the interaction. Finally, we undertook to study whether inhibitors of either the ERK or AKT cascaes affect the MEK-AKT interaction. For this purpose we CoIPed endogenous AKT by overexpressed MEK1-GFP in COS-7 cells and found that the interaction is inhibited by the PI3K inhibitor wortmannin and not by the AKT inhibitor AKTi or the Raf inhibtor PLX4032 (Fig. S6). Since PLX4032 inhibits the activatory phosphorylation of MEK, and AKTi that of AKT, it is unlikely that the activity, or activatory phosphorylation of the kinases plays a role in the interaction. The effect of wortmannin was likely due to its strong effect on Ser 298 phosphorylation. Taken together, we concluded that the MEK1-AKT interaction is direct, dependent on Ser298-MEK1 phosphorylation, but not the activatory phosphorylation of MEK and AKT and that Peptide#3 is an efficient, direct and specific inhibitor of the interaction.

### MEK-AKT interaction regulates cellular migration and adhesion

**Figure 4:**
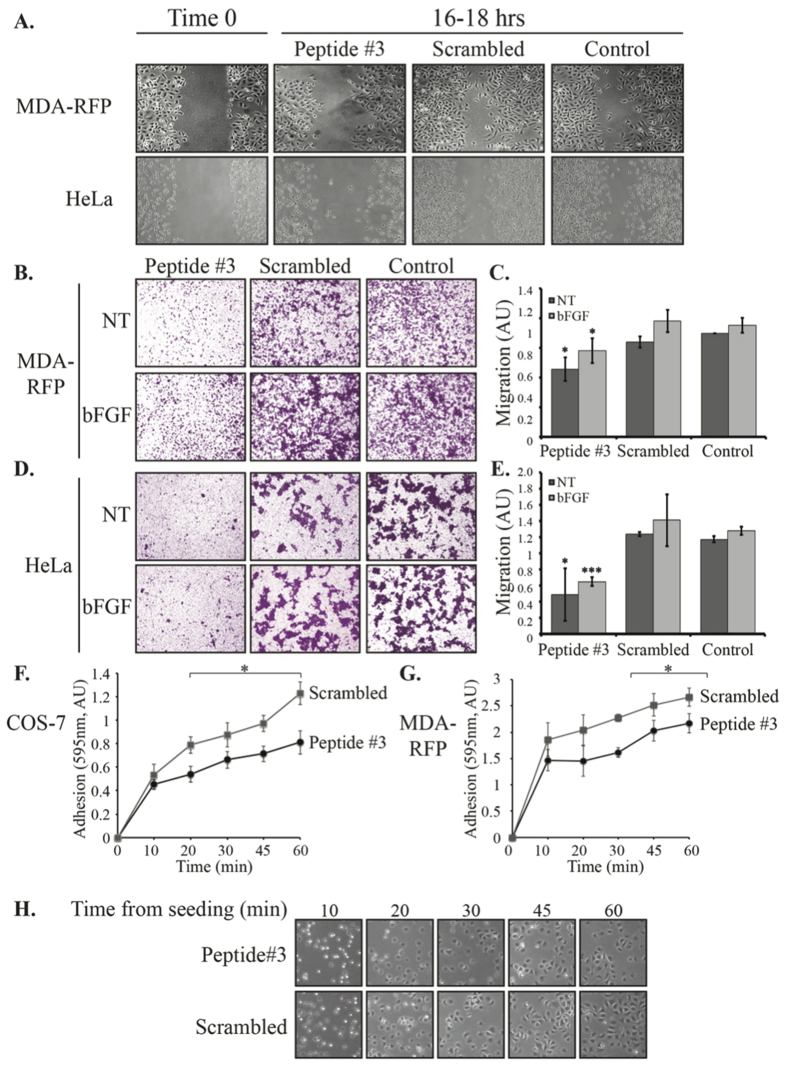
Peptide#3 affects cellular migration and adhesion. (**A**) Scratch assay of cells treated with Peptide#3. The stated cells were grown in 12× well to 80% confluence, and then a scratch was performed. The cells were washed with PBS × 1 and then allowed to grow at 1% FBS-DMEM supplemented with 10 μM Peptide#3, its scrambled control or no peptide. Wound closure was captured 16–18 hrs later. A representative picture is shown for 3 experiments performed in quadruplets. (**B–E**) Transwell assay of cells treated with Peptide#3 or controls. A suspension of MDA-RFP (**B,C**) or HeLa (**D,E**) cells was placed in the upper chamber supplemented with 10 μM Peptide#3, its scrambled control or no peptide. The cells were allowed to migrate for 16–18 hrs toward bottom chambers with or w/o bFGF, then fixed and stained with Crystal Violet. The amount of stain was quantified using ImageJ analysis and is represented in the graphs. (**F,G**) Time course adhesion of Peptide#3 treated cells. COS-7 (**F**) and MDA-RFP (**G**) cells were grown to sub-confluence, serum-starved and trypsinized. After neutralization with SBTI the cells were held in suspension for 2 hrs with Peptide#3 or its scrambled control. The cells were seeded in wells precoated with FN, and washed with PBS at the stated intervals. The remaining cells were fixed with 3% PFA and stained with methylene blue. The stain was extracted and analyzed. The graphs represent stain extract in three independent experiments. (**H**) Representative image for MDA-RFP adhesion in each time point. MDA-RFP cells were treated as described above and seeded onto cover slips precoated with FN. The cells were fixed with 3% PFA at the stated intervals and mounted on slides. Pictures were taken at x40 magnification.

After verifying that Peptide#3 specifically prevents MEK1-AKT interaction, we used it in order to elucidate the physiological importance of the interaction. Previous studies showed that Ser298 in MEK1 is involved in the regulation of cellular adhesion and motility^42^,^43^. In order to test the involvement of the interaction in these processes, the effect of Peptide#3 on wound healing was tested in both MDA-RFP and HeLa cells (Fig. 4A). Peptide#3 slowed down the wound healing process in the two cell lines, relative to scrambled controls and cells that were not treated with a peptide at all. This result indicates that the MEK-AKT interaction is important for cell migration. On the other hand, inhibiting the MEK1-AKT interaction with Peptide#3 had no effect on cellular proliferation (Fig. S5A). In order to verify this effect of the peptide, the cells tested were subjected to a transwell assay (Fig. 4B–E). Both MDA-RFP (Fig. 4B,C) and HeLa (Fig. 4D,E) cell migration were inhibited when treated with Peptide#3, but not when treated with its scrambled control or left untreated. These results confirm the role of MEK-AKT interaction in cellular migration, and suggest that Peptide#3 may inhibit not only cell migration, but possibly also invasion and metastasis.

Recollecting the effect of FN on the MEK-AKT binding, we next tested the effect of Peptide#3 on cellular adhesion and found that Peptide#3-treated COS-7 cells were less adherent in this system (Fig. 4F). The same trend was observed for MDA-RFP cells, in which cells that were treated with Peptide#3 were less adherent to FN after 30 min from seeding (Fig. 4G). A representative image of the MDA-RFP cells for each of the time points is presented in Fig. 4H. Differences between the Peptide#3-treated cells and the control scrambled peptide-treated cells is apparent already 10 min after seeding. This suggests that the effect of Peptide#3 is already manifested in the early attachment phase of the adhesion process. These observations demonstrate that Peptide#3 treatment decreases cellular adhesion, which might be, at least in part, the mechanism by which it inhibits migration.

### MEK-AKT interaction affects FoxO1 phosphorylation

**Figure 5:**
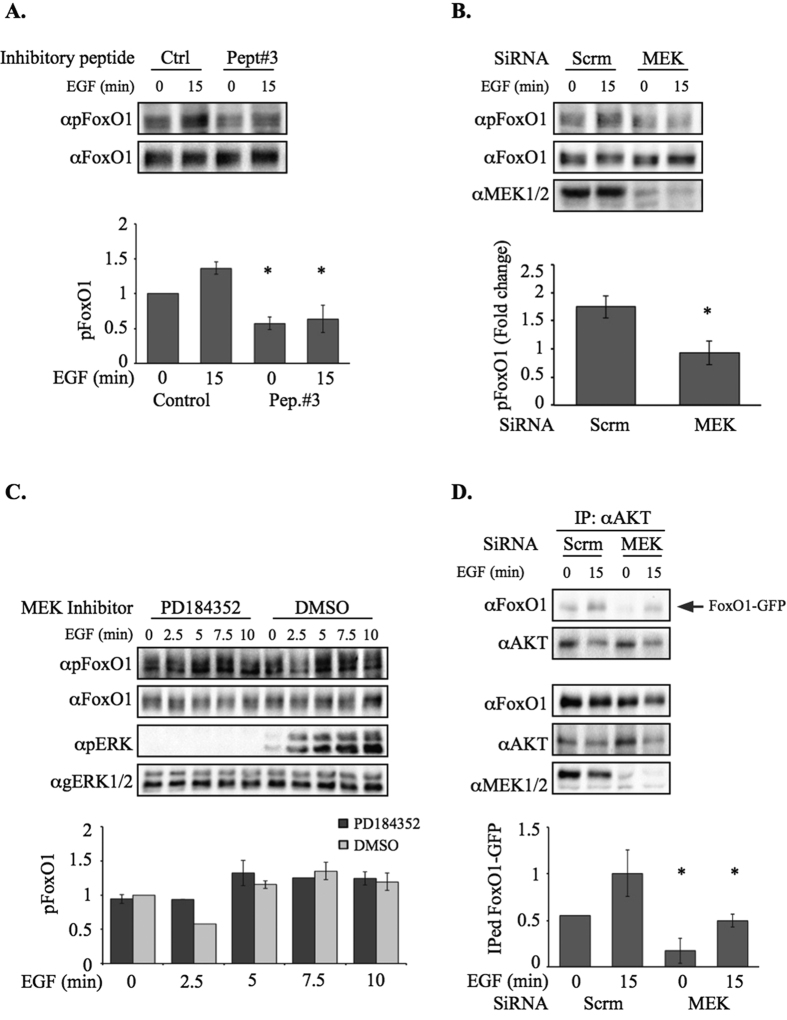
FoxO1 phosphorylation is dependent on MEK. (**A**) FoxO1 phosphorylation in the presence of either Peptide#3 or its scrambled control. HeLa cells were serum starved (0.1% FBS, 18 hrs), and then treated with Peptide #3, or its scrambled control, for 2 hrs. cells were stimulated with EGF (50 ng/ml, 15 min) or left untreated. The cytosolic extracts were subjected to Western blotting with αpS256FoxO1 or αGenFoxO1 Abs. (**B**) FoxO1 phosphorylation upon MEK silencing by siRNA. HeLa cells treated with 50 nM siRNA specific to MEK1/2 (72 hrs) were serum starved (0.1% FBS, 18 hrs), and then treated with Peptide #3, or its scrambled control, for 2 hrs. The cells were then stimulated with EGF (50 ng/ml) for 15 min or left untreated. The cytosolic extracts were subjected to Western blotting with αpS256FoxO1, αGenFoxO1 or αMEK1/2 Abs. Quantification of Foxo1 phosphorylation in fold change upon EGF stimulation. (**C**) MEK activity is not necessary for Foxo1 phosphorylation. HeLa cells were serum starved (0.1% FBS, 18 hrs), and then treated with the MEK inhibitor PD184352 (2 μM) or DMSO for 10 min. The cells were then stimulated with EGF (50 ng/ml) for the indicated times or left untreated. The cytosolic extracts were subjected to Western blotting with αpS256FoxO1, αGenFoxO1, αpTEY-ERK1/2 or αGenERK1/2 Abs. (**D**) CoIP of AKT and FoxO1 with or without MEK SiRNA. HeLa cells treated with 50 nm siRNA specific to MEK1/2 (72 hrs), and then also transiently transfected with FoxO1-GFP. When the cells reached sub-confluence they were serum starved (0.1% FBS, 18 hrs), and then either left untreated or stimulated with EGF (50 ng/ml) for 15 min. The cytosolic extracts were subjected to CoIP with αAKT Abs. The amount of interacting FoxO1-GFP was determined by Western blotting with αGenFoxO1 Ab (first panel). The same membrane was blotted with αAKT Ab (second panel). The amounts of the overexpressed proteins in the extracts as well as the effect of MEK siRNA were determined by Western Blotting (lower panels). Quantification of all experiments was done using densitometric analysis by ImageJ. The average normalized intensity is displayed, error bars represent standard error. P < 0.05 according to students *t* test.

We then undertook to identify downstream targets that are specifically affected by the MEK-AKT interaction, and not by the individual kinases. For this purpose, we treated the cells with Peptide#3 and examined the differences in protein phosphorylation of either known targets of the MEK and AKT, or of general phosphorylated proteins using both anti substrate antibodies and mass spectroscopy. The most prominent protein identified using these methods was Forkhead Box O1 (FoxO1), a transcription factor which is a known substrate of AKT on Ser256 and other sites^60^. Moreover, FoxO1 was also implicated as a key regulator of cell migration^61^, and therefore we hypothesized that this protein may be an important negative regulator of migration downstream of the MEK-AKT complex. As expected, we found that the stimulated phosphorylation of FoxO1 in our system is indeed mediated by AKT (Fig. S7A). However, peptide#3 that does not affect AKT activity (Fig. 3C) did reduce the Ser256-FoxO1 phosphorylation both before and after stimulation (Fig. 5A). These results indicate that the MEK-AKT complex is required for the phosphorylation, and led us to study the role of MEK1 within this complex in the FoxO1 phosphorylation. Thus, the knockdown of MEK1/2 (Fig. 5B), but not the inhibition of their activity by PD184352 (Fig. 5C) resulted in a significant reduction of FoxO1 phosphorylation. Moreover, we found that AKT can interact directly with FoxO1, and this interaction is reduced by MEK1/2 knockdown (Fig. 5D). This reduction in AKT-FoxO1 interaction was achieved by peptide#3 as well (Fig. S7B). Taken together, these results best fit a model in which the MEK-AKT complex is required in order to recruit FoxO1 and allow its phosphorylation by AKT within the complex. Since the complex is found in the cytoplasm, and FoxO1 is a transcription factor, it was important to follow the subcellular localization of FoxO1. Indeed, staining of the cells clearly showed that FoxO1 is localized all over the resting cells, and is restricted to the cytoplasm after stimulation (Fig. S7C). These results suggest that FoxO1 is shuttling in and out the nucleus in resting cells, but its recruitment to the MEK-AKT complex, and phosphorylation by AKT results in its cytoplasmic localization, which then allows the proceeding of migration, but does not affect proliferation.

### MEK-AKT interaction is a possible therapeutic target for metastasis

**Figure 6:**
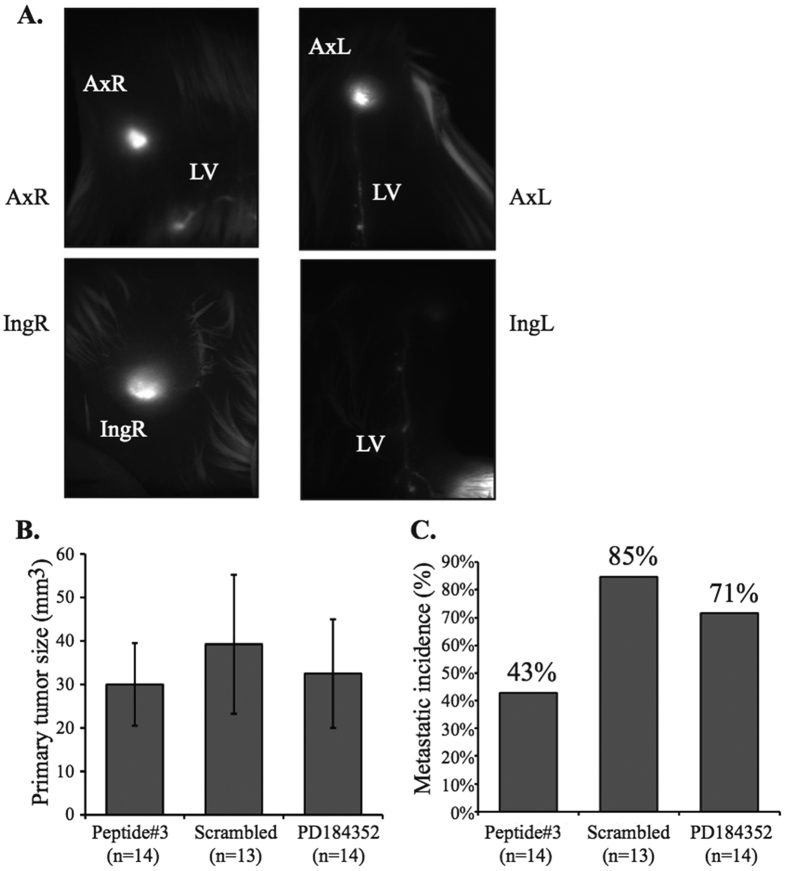
Peptide#3 treatment in mouse metastatic models. **(A)** Representative image of lymph node metastasis. Female *nude* mice were inoculated with 1.5 × 10^6^ harvested MDA-MB-231-RFP cells. A representative picture is shown for Ax = Axillary, Ing = Inguinal, LV = Lymph vessel, PT = Primary tumor. (**B**) PT volume was measured with caliper once a week. Graph represents average PT size at experiment end-point in two independent experiments. (**C**) Metastatic onset. Nude mice were tested with a fluorescent microscope for the incident of metastasis either in the ipsilateral or contralateral lymph nodes. Graph represents the percentage of mice that developed metastasis in each of the treatment groups in two independent experiments.

The ERK cascade and the PI3K pathway play a role in most cancers^62^. Finding that inhibition of MEK1-AKT interaction by Peptide#3 decreased cellular migration and adhesion, but not proliferation, we set out to explore Peptide#3 as an anti-metastatic drug in a mouse model. We utilized a previously described model^63^,^64^, in which a primary breast tumor (PT) metastasizes to the mouse lymph nodes (Fig. 6A). MDA-RFP cells were injected to *nude* mice and primary tumors were allowed to develop for approximately 7 days. The mice were then randomly selected for one of three treatment groups: (1) *Peptide#3*, (2) *Scrambled peptide*, and (3) *Control* (PD184352), which was used here to show whether the effectis downstream of ERK activity. The treatments were administrated *i.v.* three times a week, for 4 weeks. At the end point of the experiment there were no significant differences in the PT size for the three different treatment groups (Fig. 6B). Then, we examined the metastasis formed at nearby lymph nodes and found that *nude* mice that were treated with Peptide#3 had significantly less incidence of metastasis. Only 43% of the treated mice relative to 85% in the scrambled control-treated group, and 71% in the MEK inhibitor treated group. These results suggest that unlike MEK and AKT inhibitors, the inhibition of the MEK1-AKT interaction may be used as a new therapeutic agent to reduce the emergence of breast metastasis.

## Discussion

**Figure 7:**
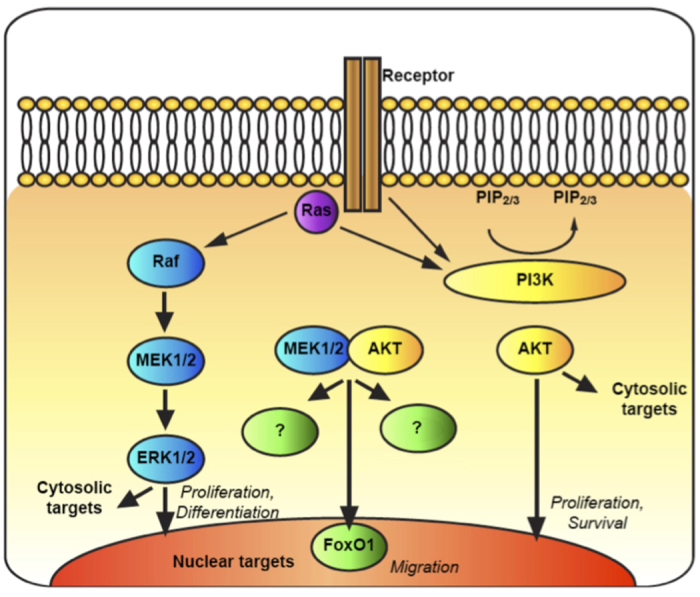
Schematic representation of the results.

The ERK cascade and the PI3K/AKT pathway are two major signaling pathways that regulate many different and even opposing cellular processes including proliferation, differentiation and survival, and in rare cases also apoptosis. Crosstalk between the ERK cascade and other signaling pathways is one of the means by which it directs its signaling specificity. There are several known intersections between the ERK cascade and the PI3K/AKT pathway, which control and regulate a broad spectrum of processes. Here we studied a novel physical interaction between MEK1/2 and AKT, and elucidated its physiological role, discovering a new signalling route, different from those of either ERK cascade or PI3K/AKT pathway (schematically represented in Fig. 7). Our initial experiments were designed to demonstrate the interaction between the two MEK isoforms, MEK1 and MEK2, and the three AKT isoforms. In these experiments the interaction of MEK1 and MEK2 with AKT was confirmed using several distinct methods, including (*i*) CoIP of overexpressed MEK isoforms with Abs for endogenous AKTs, (*ii*) the reciprocal CoIP of overexpressed AKT isoforms with Abs for endogenous MEKs, (*iii*) CoIP of endogenous proteins, (*iv) in vitro* binding assay and (*v*) PLA. These methods clearly showed that MEK can interact with AKT, and since the results were reproduced in several cell lines, it is likely that this interaction can be found in the majority of cells.

The interaction between MEK1/2 and AKT increases in response to mitogenic stimulation with EGF. This stimuli-dependent effect on binding between the two proteins suggests the interaction is part of a regulatory network that governs and conduits the ultimate cellular response. It is extremely likely that the interaction between MEK and AKT is direct, both before and after stimulation. This is because of the two recombinant MEK isoforms interacted in an *in vitro* assay with AKT which was IPed using stringent washes, and also because of the recognition in a PLA assay (Fig. 1) that indicates proximity of less than 50 Å (while smallest scaffold proteins are approximately 45 Å). The kinetics of interaction between MEK and AKT was highly correlated to the phosphorylation of Ser298 in MEK1, but not to the phosphorylation of its activation loop Ser218/Ser222. This residue’s adhesion-dependent phosphorylation was shown to promote MEK1’s activation by mitogenic stimulation, and was suggested to do so by modulating the physical and functional interactions between Raf, MEK and ERK^42^. Our results however, may indicate that the story is not so simple, as AKT may be involved in this process as well. Since MEK2 also interacted directly with AKT, we set out to locate a corresponding residue that can regulate the interaction of MEK2 and AKT. Thus, MEK2’s Ser306 was identified as a MEK1-Ser298 analougus phosphorylation site, and its phosphorylation regulates the MEK2-AKT interaction. These phosphorylations contribute much of the binding energy, but other adjecent residues in the PRD are important as well (Fig. S4).

In this study we identified the region on MEK1 and MEK2 that interact with AKTs. This allowed us to design a competing peptide (Peptide#3, AAs: AETPPRPRTPGRPLS). This peptide nicely compete with the interaction of MEK1 and AKTs, probably due to its interaction or masking of the binding site in AKT. Therefore, the sequence of this peptide may provide a clue regarding the binding site in AKT. To look into this point, we utilized ANCHORSmap calculations, which is a computational protein-protein docking program, taking into account that AKT2 is the predominant binding isoform of MEK1. Mapping some of the unique residues of AKT2, that are distinct from that of AKT1 and AKT3, that shows them to form an elongated L-shaped cavity on the surface of AKT2 (residues 268–269 and 302). This region as well as additional residues may provide a Low ΔG (kcal/mol) anchoring spots for Peptide#3. Importantly, this interaction does not seem to interfer with the catalytic pocket of AKT2, which is consistent with our observation that the activity of AKT is not required or affected either by the binding to MEK1 or to the peptide (Fig. 2 and data not shown). However, much more work is required in order to verify the identity of the binding site in AKTs.

It was important to elucidate the role of MEK-AKT interaction. In order to do so we designed and utilized Peptide#3, which corresponds to amino acids 283–298 in the PRD of MEK1, and found that prevention of the interaction inhibits cellular migration and adhesion in various cell types. We also found that the MEK-AKT complex lead to phosphorylation of FoxO1, a transcription factor that is a known regulator of cell migration^61^. This phosphorylation required both the presence of MEK and the activity of AKT, and thus represents a downstrean effect that is specific to the MEK-AKT interaction, and not to either of the two more characterized pathways of these proteins. FoxO1 was previously reported to localize mostly to the nucleus of starved CV1 cells and exported out of that location after stimulation^65^. This export has been reported to release the inhibtory effect of FoxO1 on cell migration and allow an enhanced cell motility^61^,^66^. This export and the cytoplasmic accumulation was suggested to be regulated by AKT phosphorylation. Our results generally agree with this suggestion, but the fact that FoxO1 is seen all over in resting cells may suggest that it is constantly shuttling in and out of the nucleus. The MEK-AKT complex formation allows the recruitmnet of FoxO1 to the cytosolic complex, and FoxO1’s phosphorylation by AKT, which fixes its cytoplasmic localization and allows migration to proceed. The MEK-AKT interaction might affect other downstream effectors, but more work is needed in order to complete the mapping of the signalling resulting from this interaction.

Previous publications clearly demonstrated that the ERK cascade and the PI3K pathway play a role in most cancers^62^. Components of these pathways are frequently mutated, activated or aberrantly expressed in many human cancers. However, consequences of their activation in tumors are not fully understood, and single signaling pathway inhibition favors the emergence of resistance mechanisms. Consequently, it is now commonly believed that better cancer treatment can be achieved by using MEK inhibitors in combination with PI3K inhibitors, due to a feedback mechanism between the two pathways^52^. An increasing body of research is trying to exploit the currently known intersections between the two pathways in order to develop efficient anti-cancer therapies^58^,^67^,^68^. However, the majority of these treatments target the activity of a given signaling component or components, and not the context in which this component acts, such as protein-protein interactions. In contrast, our peptide described here does affect the interaction and not the activity, and therefore, may have milder inhibtor affect on specific metastasis-related processes. While average primary tumor size was not significantly changed by treatment with Peptide#3, the incidence of metastasis was significantly lower in *nude* mice that were treated with Peptide#3 compared to mice that were treated with the scrambled peptide or the MEK inhibitor PD184352. This result suggests the MEK-AKT interaction as a promising novel therapeutic target in treatment of cancer and prevention of metastasis.

In summary, we show for the first time that MEK1 and MEK2 interact with AKT and that this interaction participates in the regulation of cell migration and adhesion. Our results best fit a model in which at basal state, when both MEK and AKT are inactive, there is little interaction between the two. Once phosphorylation of MEK1’s Ser298/MEK2’s Ser306 occurs, by addition of mitogenic or adhesion stimulants, it increases the MEK1/2-AKT interaction. This interaction plays a role in the regulation of cell migration and adhesion, since its inhibition hinders both. It is conceivable that this phosphorylation leads to a conformational change in the PRD of MEK, exposing the nearby interaction site with AKT. Lastly, the MEK-AKT interaction suggests a novel and unique therapeutic target for anti-breast cancer therapies, since treatment with Peptide#3 hinders the onset of metastasis.

## Materials and Methods

### Reagents

The MEK inhibitor PD184352, the AKT inhibitor AKTi, Epidermal Growth Factor (EGF), basic Fibroblast Growth Factor (bFGF), Polyethylenimine (PEI), Avidin-FITC and 4′6-diamino-2-phenylindole (DAPI) were purchased from Sigma-Aldrich (Rehovot, Israel). Protein A/G PLUS-agarose beads were obtained from Santa Cruz Biotechnology, Inc. (CA, USA). Glutathione beads were purchased from GE-healthcare and Proximity ligation assay kit was from Olink Bioscience (both Uppsala, Sweden). Albumin bovine serum (BSA) was purchased from MP biomedical (OH, USA). Si RNAs and Dharmafect were from Thermo Fisher Scientific (CO, USA). NBT/BCIP developing substrates were purchased from Promega (Wis, USA). Soybean trypsin inhibitor (SBTI) was purchased from Biological Industries (Beit HaEmek, Israel).

### Antibodies

αGenAKT, αpERK1/2(pTEY-ERK), αGenERK1/2 and αGenMEK1/2 Abs were from Sigma-Aldrich (Rehovot, Israel). αMEK1(H8), and αMEK1(C18) Abs were from Santa Cruz Biotechnology (CA, USA). αpT308AKT, αpS473AKT, αpS218/222MEK, αGenMEK2, αpS256FoxO1 and αGenFoxO1 Abs were from Cell Signaling Technology (Boston, MA). αpS298MEK1 Ab was from AbCam (Cambridge, England). αGFP Ab was from Roche Diagnostics GmbH (Mannheim, Germany). αpS306MEK2 Phosphosite-specific Abs for the S306-phosphorylated form of MEK2 were prepared by the Antibody Unit of the Weizmann Institute of Science. The rabbit Abs were generated against a sequence corresponding to residues 298–311 of the human MEK2 that contained a phosphorylated Ser residue: C-PRPPGRPV(pS)GHGMD. Rabbit serum Abs were affinity purified against their immunizing peptide as well as against the non-phosphorylated peptide using the SulfoLink kit (Thermo Scientific, Rockford, IL).

### Peptides

Custom peptides overlapping MEK1 sequence were ordered from Peptide 2.0 (Virginia, USA): peptide #1 (corresponding residues 253–268) PPPDAKELELMFGCQ, peptide #2 (263–288) MFGCQVEGDAAETPP, peptide #3(283–298) AETPPRPRTPGRPLS, peptide #4 (293–308) GRPLSSTGMDSRPPM and a scrambled peptide PLRTPGSPRARPETP. Peptides used in cell assays were adjoined myristic acid (Myr) on their N-terminus.

### DNA Constructs and mutations

MEK1/2 were cloned in pEGFP-N1 and AKT1/2/3 were cloned in pEGFP-C1 (Clontech, Mountain View, CA). Point mutations were performed by site-directed mutagenesis using specific primers and confirmed by DNA sequencing. GST was expressed from pGEX41 (GE healthcare, Buckinghamshire, UK). MEK1/2 were cloned in pGJ vector (Rehovot, Israel) and flanked by SpeI and NotI restriction sites for the recombinant proteins tagged by GST and 8xHis.

### Cell Culture and transfections

HeLa and COS-7 cells were cultured in Dulbecco’s modified Eagle’s medium (DMEM) supplemented with 2 mM L-glutamine, 1% Pen/Strep and 10% fetal bovine serum (FBS). MDA-MB-231-RFP (clone #3) cells were generously provided by Prof. Avigdor Scherz, the cells were cultured in RPMI 1640 medium with 2 mM L-glutamine, 1% Pen/Strep, 10% FBS, 1% NaPyr and in the presence of 250 μg/ml hygromycin. All cells were maintained at 37 °C in a humidified atmosphere of 95% air and 5% CO2. Cells were transfected with DNA construct using PEI at 30% confluence and 10% FBS.

### Immunofluorescence microscopy

Cells were grown to 70% confluence on cover-slips as previously described^69^. The cells were fixed in 3% paraformaldehyde (PFA) in PBS (20 min, 23 °C), and incubated with 2% Albumin bovine serum (BSA) in PBS (15 min, 23 °C), followed by permeabilization with Triton X-100 (0.1% in PBS, 5 min, 23 °C). The fixed cells were then incubated with the primary Abs (60 min, 23 °C), washed three times with PBS and incubated with rhodamine or Alexa Fluor 568-conjugated secondary Ab (60 min, 23 °C), and DAPI. Slides were visualized by fluorescence microscope (Olympus BX51, x40 magnification). Background correction, and contrast adjustment of raw data images were performed using Photoshop (Adobe, CA, USA).

### Preparation of cell extracts and western blotting

Cells were grown to subconfluence and serum-starved for 16 h (0.1% FBS). After treatments, the cells were rinsed twice with ice-cold PBS and once with ice-cold Buffer A, scraped into Buffer H and disrupted by sonication (60 W, 2 × 6 sec) on ice. The extracts were centrifuged (15,000 rpm, 15 min at 4 °C) and the supernatants were either subjected to co-immunoprecipitation or resuspended and boiled for 5 min in sample buffer. The samples were then subjected to 12% SDS-PAGE and Western blotting with the appropriate Abs, which were detected using alkaline phosphatase or ECL according to the manufacturer’s instructions. A representative western blot of at least three independent experiments is shown for all cases.

### Co-Immunoprecipitation

Cell extracts were produced as described above and incubated for 2 hours (4 °C, with rotation) with A/G-agarose beads pre-linked with specific Abs (1 hr, 23 °C). The bound A/G beads were washed three times with ice-cold CoIP washing buffer. The washed beads were then resuspended with 1.5X sample buffer and boiled.

### *In-vitro* interaction

Cell extracts were produced as described and incubated for 2 hrs (4 °C) with A/G-agarose beads (Santa Cruz Biotechnology) pre-linked with specific Abs (1 hr, 23 °C). The bound A/G beads were sequentially washed once with RIPA buffer, then twice with 0.5 M LiCl and twice with Buffer A. The bound A/G beads were then resuspended in Buffer A containing 0.01% BSA and aliquoted. GST tagged proteins were incubated with the beads 2 hrs (4 °C) then washed and resuspended in 1.5 X sample buffer and boiled.

### Proximity ligation assay (PLA)

Protein–protein interactions were detected with Duolink PLA Kit (Olink Bioscience), as previously reported by our laboratory^70^. Briefly, cells were grown, fixed and permeabilized as described. The samples were then incubated with primary Abs against two examined proteins (60 min, 23 °C), washed, (0.01 M Tris HCl pH 7.4, 0.15 M NaCl and 0.05% Tween 20), and then incubated with specific probes (60 min, 37 °C), following by DAPI staining to visualize nuclei and wash with Buffer B: 0.2 M Tris HCl pH 7.5, 0.1 M NaCl. The signal was visualized as distinct fluorescent spots by fluorescence microscope (Olympus BX51, x40 magnification). Background correction, contrast adjustment of raw data images and the quantification of the fluorescence signal were performed using Photoshop (Adobe) and ImageJ.

### Protein purification

GST, GST-MEK1-8xHis and GST-MEK2–8xHis were expressed in E. coli BL21(DE3)pLysS. Bacterial cells were grown at 30 °C to OD600 nm = 0.5, and the expression of recombinant proteins was induced by 0.5 mM IPTG for 4 to 5 hours. Pelleted bacterial cells were lysed, and purification of recombinant proteins was performed using first glutathione affinity chromatography (GE Healthcare, Buckinghamshire, UK) and then ion metal affinity chromatography with Ni-NTA His-Bind resin (Novagen, Darmstadt, Germany) according to the manufacturer’s protocol. Elutions were dialyzed against PBS (16 hrs, 4 °C), and the amount of purified protein was detected by Bradford method.

### Cell adherence to FN

Cells were grown to subconfluence, serum-starved (0.1% FBS for 16 hrs) and then harvested by trypsinization. The trypsin was inactivated by addition of soybean trypsin inhibitor (SBTI, 100 μg/ml), and cells were collected by centrifugation, resuspended in starvation DMEM, and held in suspension for 2 hrs at 37 °C (0.5 × 106 cells/ml). Cell culture dishes were precoated (30 min, 37 °C) with FN (10 μg/ml). Suspended cells were distributed onto the coated dishes (0.5 × 106 cells/well in 12-well, 8 × 106 cells/plate in 10 cm) and incubated at 37 °C for various times following plating. The attached cells were rinsed in PBS and then either fixed in 4% paraformaldehyde (2 hrs, 23 °C) or harvested for CoIP assays. Fixed adherent cells were assessed using methylene blue assay, they were washed once in 0.1 M sodium borate buffer (pH 8.5), and thereafter incubated with 1% methylene blue for 10 min. Excess stain was washed out with double distilled water. Stain was extracted with 0.1 M HCl, and OD595 nm was determined in an ELISA reader.

### *In Vitro* Migration Assays

Transwell Assay: The migration of HeLa and MDA-MB-231-RFP cells was assayed in 24-well Transwell plates with polycarbonate filters of 8 μm pores (Corning, NY). A suspension of cells (5–6 × 104 cells/200 μl) was placed in the upper chambers. The lower chambers were filled with 600 μl of the corresponding medium with or w/o 20 ng/ml bFGF. Cells were allowed to migrate for 16–18 hrs in the presence of different treatments. Thereafter, cells were removed from the upper compartment of the filter with a cotton swab. Cells that reached the lower surface of the filter were fixed with 3% paraformaldehyde, stained with 0.3% crystal violet, and images were then captured using a digital camera coupled with a microscope. Relative migration was quantified following stain extraction with methanol and a 540 nm measurement in an ELISA reader. “Wound Healing” Assay: COS-7, HeLa and MDA-MB-231-RFP cells were grown to 90–95% confluence. Thereafter, a cell scratch spatula was used to create a fixed-width wound in a cell monolayer, followed by incubation with medium containing 1% FBS with or w/o the presence of peptides. Wound closure was monitored after 16–18 hrs.

### Animal studies

All animal experiments were approved and carried out in “accordance” with the approved guidelines of the Animal Care and Use Committee of the Weizmann Institute of Science (Rehovot, Israel). Female CD-1 nude (HsdHli:CD1-Foxn1nu) mice (Harlan), 6 weeks of age, were inoculated *s.c.* to fat pad with 1.5 × 10^6^ MDA-MB-231-RFP cells in 100 μl PBS. Tumors were allowed to develop to the size of ~100 mm^3^ and then animals were randomly allocated to different treatment groups. Peptide#3, its scrambled control (both at 5 mg/kg concentration) and the MEK inhibitor PD184352 (at 2.5 mg/kg) were administered by injection *i.v.* into the tail vein, three times a week, for four weeks. To assess any signs of systemic toxicity, body weight was monitored three times per week as well.

### Statistical Analysis

Data are expressed as mean ± S.E. Statistical evaluation was carried out using functional analysis and Student’s *t* test (two-tailed) to test for differences between the control and experimental results. Values of p < 0.05 were considered statistically significant.

## Additional Information

**How to cite this article**: Procaccia, S. *et al*. Direct binding of MEK1 and MEK2 to AKT induces Foxo1 phosphorylation, cellular migration and metastasis. *Sci. Rep.* **7**, 43078; doi: 10.1038/srep43078 (2017).

**Publisher's note:** Springer Nature remains neutral with regard to jurisdictional claims in published maps and institutional affiliations.

## Supplementary Material

Supplementary Figures
