# Supplementary material for: Direct binding of MEK1 and MEK2 to AKT induces Foxo1 phosphorylation, cellular migration and metastasis
Source: Sci Rep. 2017 Feb 22;7:43078. doi: 10.1038/srep43078 (PMC5320536; doi:10.1038/srep43078)
Supplement: Supplementary Figures [file srep43078-s1.pdf]

Supplimentary figures

Direct binding of MEK1 and MEK2 to AKT induces Foxo1 phosphorylation, cellular migration and metastasis

Shiri Procaccia, Merav Ordan, Izel Cohen, Sarit Bendetz-Nezer and Rony Seger

Department of Biological Regulation, The Weizmann Institute of Science,

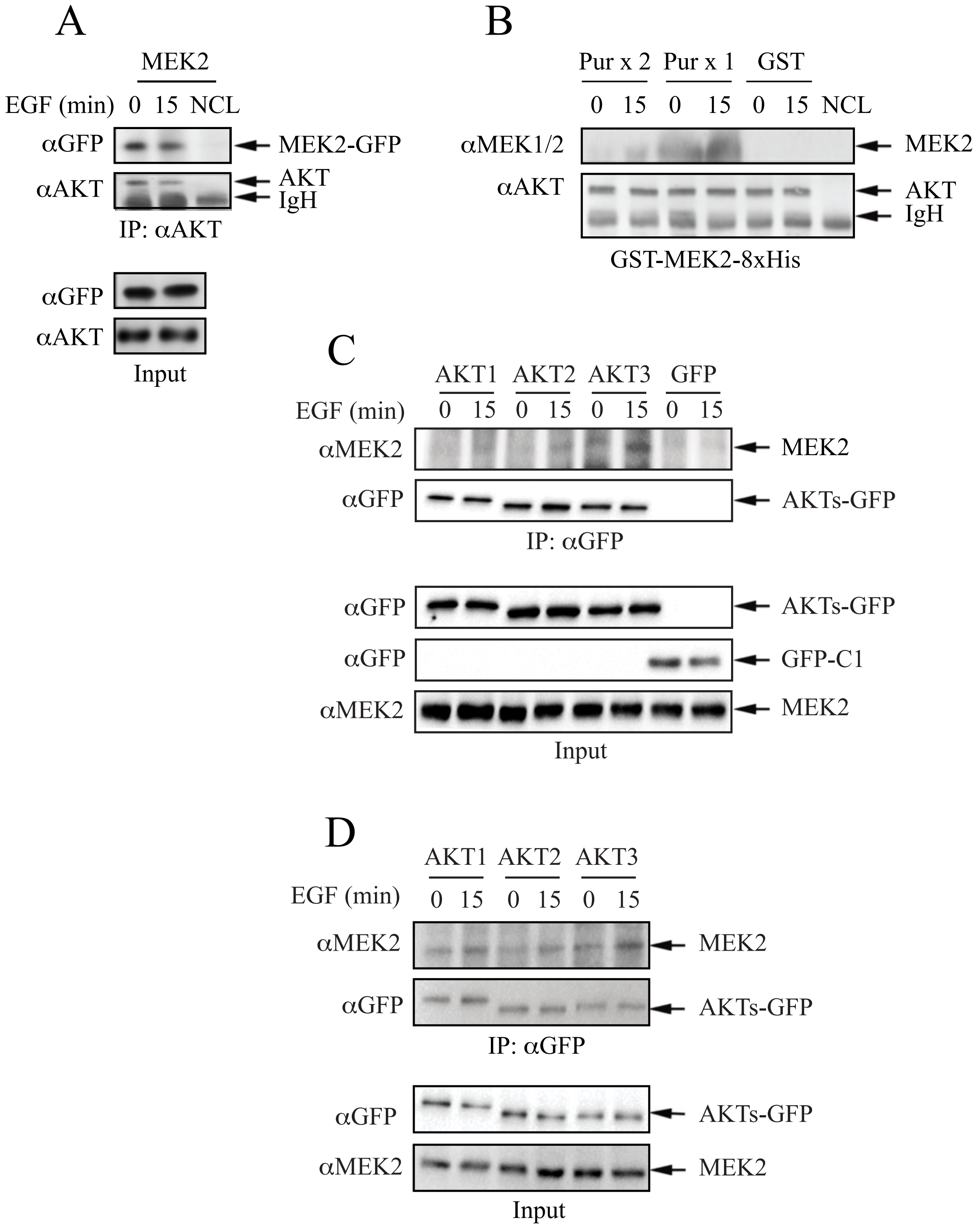

Fig. S1. MEK2 interacts directly with AKT. (**A**) Overexpressed MEK2 interacts with AKT in $MEF^{MEK1-/-}$ cells. AKT was IPed from $MEF^{MEK1-/-}$ cells that were transiently transfected with MEK2-GFP plasmid. When the cells reached sub-confluence they were serum starved (0.1% FBS, 18hrs), and then either stimulated with EGF (50 ng/ml, 15min) or left untreated. The cytosolic extracts were subjected to CoIP with αAKT Abs. The amount of interacting MEK2-GFP was determined by

Western blotting with αGFP Ab. The same membrane was blotted with αAKT Ab. The amounts of the MEK2-GFP and AKT in the extracts were determined by Western blotting with αGFP and αAKT Abs (*bottom panels*). (**B**) *In vitro* interaction of IPed AKT and recombinant MEK2. AKT was IPed from HeLa cells treated as described in A by sequential washes once with RIPA buffer, then twice with 0.5 M LiCl and twice with Buffer A. Recombinant GST-MEK2-8xHis that was purified by GST column (pur×1, 500 ng/sample), or by GST column and then NiNTA column (pur×2, 200 ng/sample), or GST alone control (500 ng/sample) were incubated with the bound AKT in the presence of 0.1% BSA to avoid non-specific interactions. Interaction was detected by Western blotting with αMEK1/2 Abs. The equal amounts of AKT in the IP samples were determined by Western blotting with αAKT Ab. (**C**) CoIP of MEK2 with overexpressed AKT1/2/3 in COS7 cells. COS7 cells were transfected with AKT1-GFP, AKT2-GFP, AKT3-GFP and GFP control plasmids, grown for 36 hours serum-starved for 14 hrs and then either treated with EGF (50 nM 15 min), or left untreated. Cytosolic extracts of these cells were subjected to CoIP with αGFP Ab. The amounts of interacting AKTs and loaded MEK2 were determined by the indicated Abs. **(D)** CoIP of MEK2 with overexpressed AKT1/2/3 in MEK1 KO MEFs. MEK1-KO MEFs were transiently transfected with AKT1-GFP, AKT2-GFP, AKT3-GFP plasmids and treated as in C. The cytosolic extracts of either EGF stimulated or untreated cells were subjected to CoIP with αGFP Ab. The amounts of interacting AKTs and loaded MEK2 were determined by the indicated Abs.

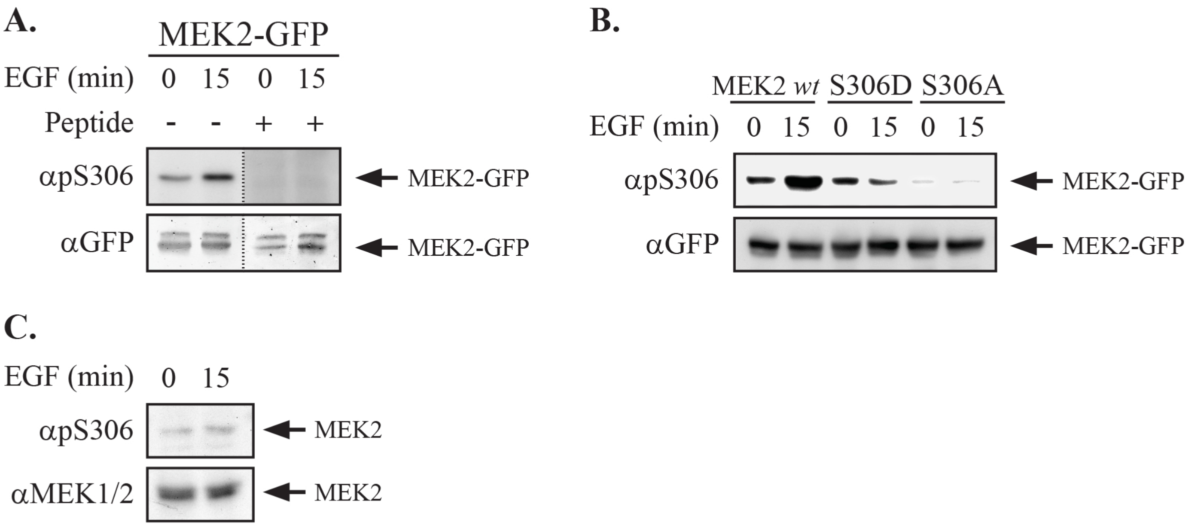

Fig. S2. Newly developed Ab to pSer306 identifies it as a new MEK2 phosphorylation site. (**A**) Competing the αpS306 Ab with the antigenic peptide. HeLa cells were transiently transfected with MEK2-GFP plasmid. When the cells reached sub-confluence they were serum starved (0.1% FBS, 18hrs), and then either stimulated with EGF (50 ng/ml, 15min) or left untreated. The cytosolic extracts were immunoblotted with αpS306 in the absence or presence of the antigenic peptide (200 ng/ml). The same membranes were blotted with αGFP in order to evaluate the total MEK2-GFP amount. (**B**) αpS306 Ab does not recognize site-specific mutants. HeLa cells were transiently transfected with MEK2-GFP *wt* or its mutants S306D and S306A. The cells were treated as in A and the cytosolic extracts were immunoblotted with αpS306 or αGFP Abs. (**C**) αpS306 Ab recognizes the endogenous MEK2 protein phosphorylation. $MEF^{MEK1-/-}$ cells were serum starved and then either stimulated with EGF (50 ng/ml, 15min) or left untreated. The cytosolic extracts were immunoblotted with αpS306 or αGFP Abs. (**D**) αpS306 Ab recognizes phosphorylation only in MEK2. HeLa cells were transiently transfected with MEK1-GFP or MEK2-GFP plasmids, and treated as in A. The cytosolic extracts were immunoblotted with αpS306, and then the same membrane was blotted with αGFP in order to evaluate the total overexpressed protein amount.

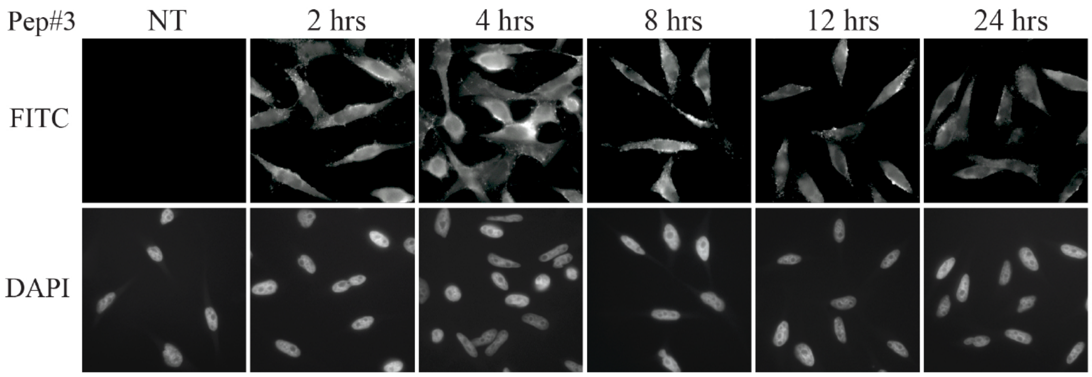

Fig. S3. Intracellular distribution and stability of myristolated Peptide#3. HeLa cells were grown on cover slips to 70% confluence, serum starved (0.1% FBS, 18hrs), and treated with Peptide#3 conjugated with myristic acid on its N-term and biotin on its C-term for the indicated times. Cells were fixed with 3% PFA and stained with Avidin-FITC and DAPI. The peptide was visualized using a fluorescent microscope (×40 magnification).

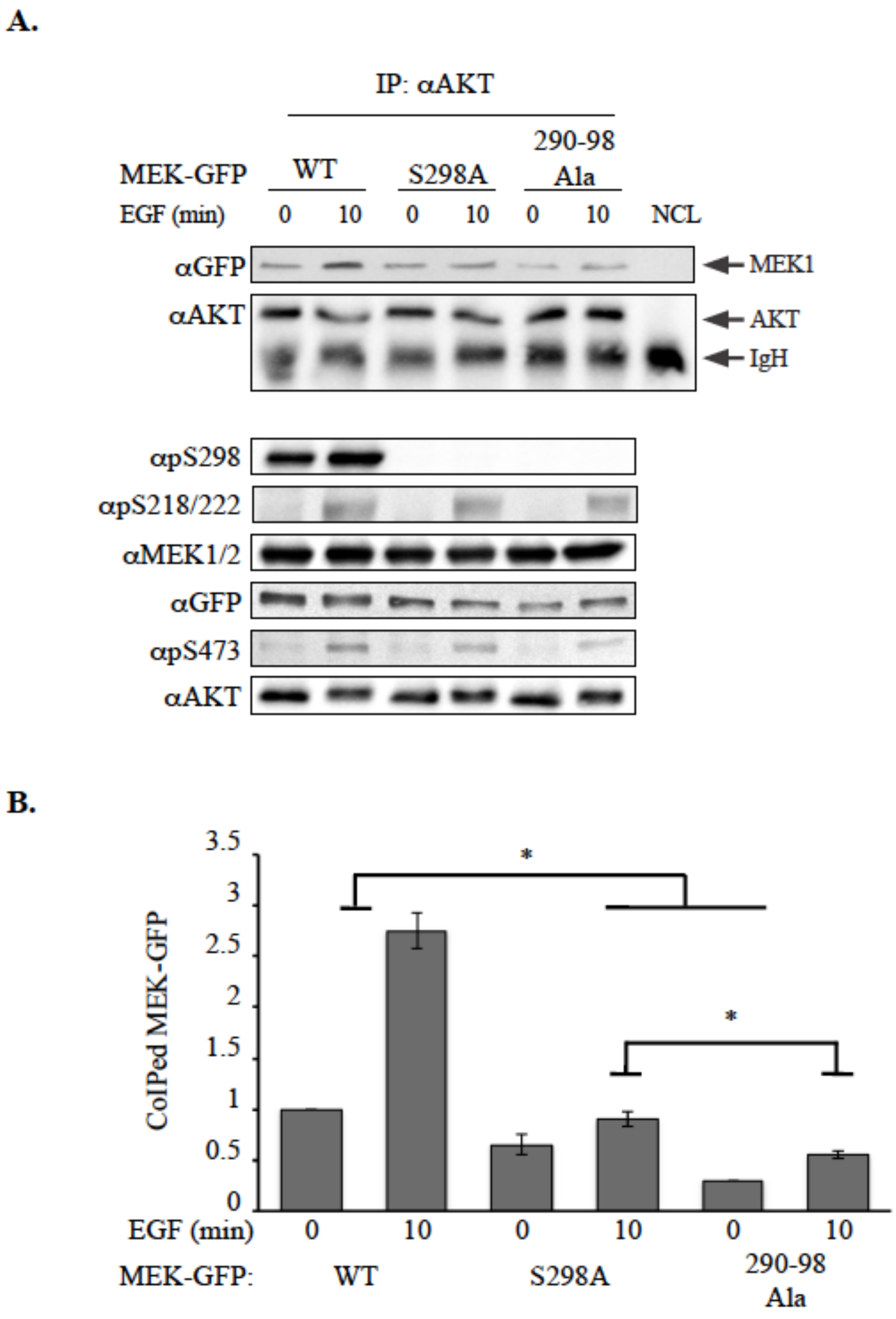

Fig. S4. Amino acids 290-297 in the MEK1 PRD domain add to the interaction with AKT. (**A**) AKT interaction with *wt* or mutated MEK. COS-7 cells were transiently transfected with MEK1-GFP *wt* or its mutants S298A and 290-98Ala. When the cells reached sub-confluence they were serum starved (0.1% FBS, 18hrs), and then either left untreated or stimulated with EGF (50 ng/ml) for 10 min. The cytosolic extracts were subjected to CoIP with αAKT Abs. The amount of interacting MEK1-GFP was determined by Western blotting with αGFP Ab (first panel). The same membrane was blotted with αAKT Ab (second panel). The amounts of the overexpressed proteins in the extracts as well as the phosphorylations of MEK1's Ser298 and Ser218/222 and of AKT's Ser473 were determined by Western Blotting (lower panels). (**B**) Quantification of MEK1-AKT interaction upon EGF stimulation. Western blots were subjected to densitometric analysis by ImageJ. The average normalized MEK1-AKT interaction is displayed, error bars represent standard error. $P<0.05$ according to students T test.

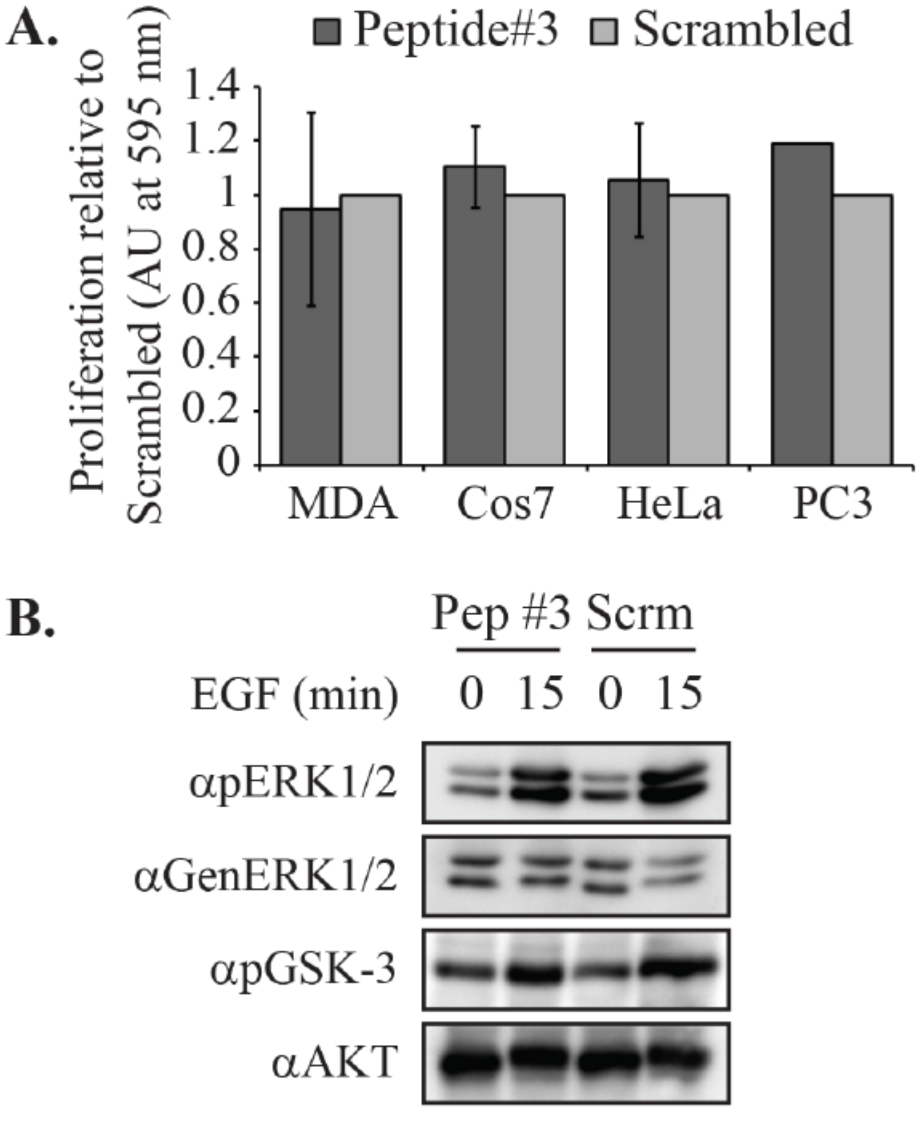

Fig. S5. No effect of Peptide#3 on cellular proliferation. (**A**) Peptide#3 does not affect cellular proliferation. MDA-RFP, COS-7, HeLa or PC3 cells were seeded in 12 ×well at 10% or 20% (for PC3) confluence in 10%FCS-DMEM. Four hrs later the media was changed to 1%FCS-DMEM together with 10μM of Peptide#3 or its scrambled control. Twenty-four hrs later another boost of peptide was added, and after additional 24 hrs the cells were washed with PBS×1, fixed with 3% PFA and stained with methylene blue as described under material and methods. Graph represents at least three independent experiments. (**B**) Peptide#3 does not affect ERK and GSK-3. COS-7 cells were serum-starved (0.1% FBS, 16 hrs) at sub-confluency and pre-incubated with Peptide#3 or its scrambled control for 2 hrs. The cells were then stimulated with EGF (50 ng/ml) for 15 min or left untreated, and harvested with RIPA. The cytosolic extract were subjected to Western blotting with the indicated Abs.

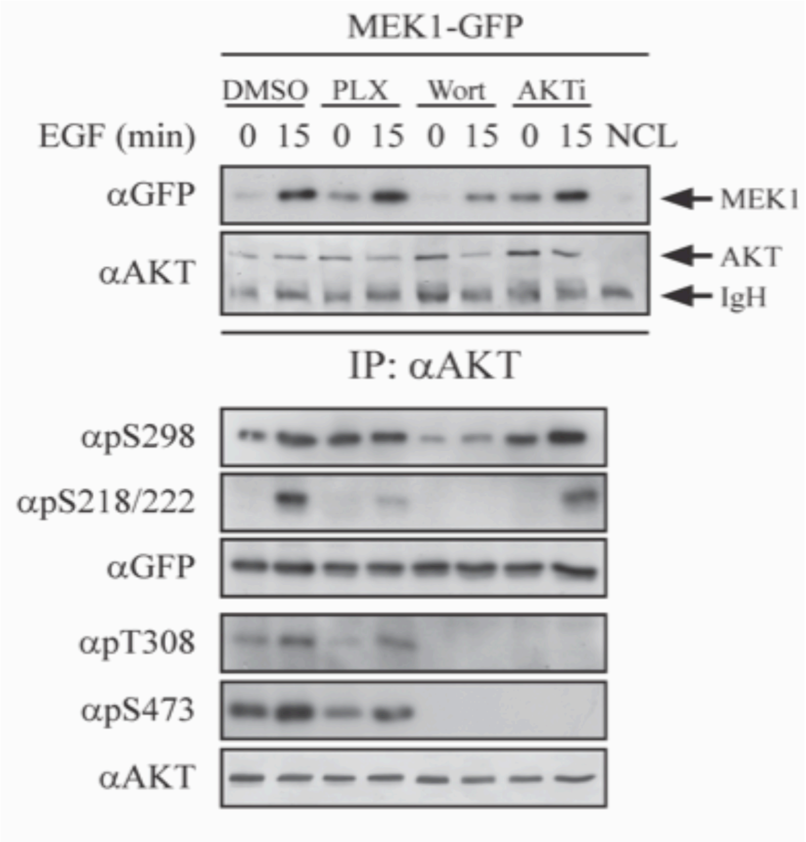

Fig. S6. CoIP of MEK1 by AKT with kinases inhibitors. COS-7 cells were transiently transfected with MEK1-GFP. Sub-confluent cells were serum starved (0.1% FBS, 16hrs), added the indicated kinase inhibitor for 20 min, and then stimulated with EGF for 15 min or left untreated. The cytosolic extracts were subjected to Western blotting with the indicated Abs. (*lower* panels) and to CoIP with αAKT Ab (*upper panels*). The amount of interacting MEK1-GFP was determined by Western blotting with αGFP Ab. The same membrane was blotted with αAKT Ab.

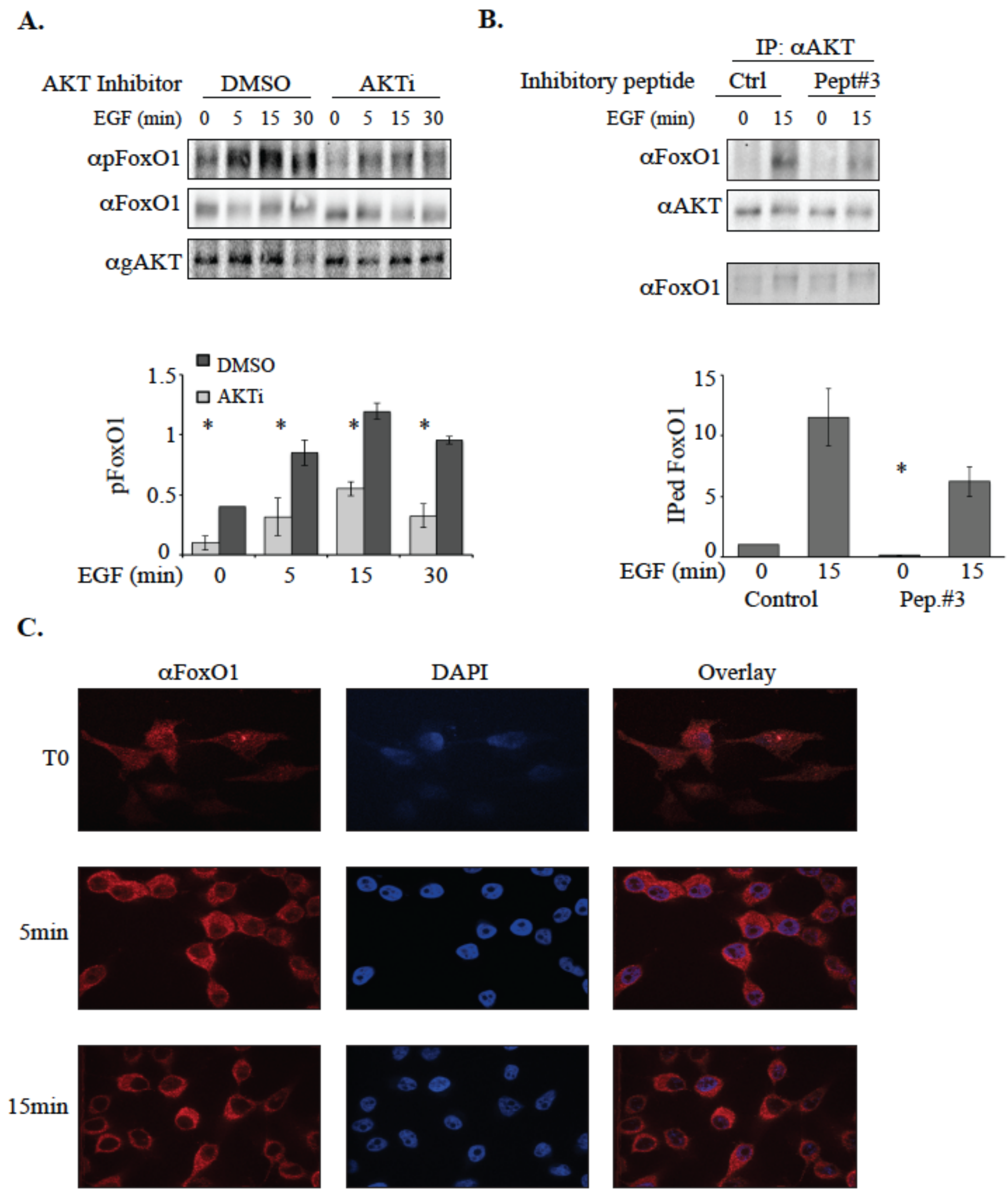

Fig. S7. Effect of AKT and MEK1/2 on Foxo1 phosphorylation and localization. (**A**) AKT activity is needed for FoxO1 phosphorylation. HeLa cells were serum starved (0.1% FBS, 18hrs), and then treated with the AKT inhibitor AKTi (1μM) or DMSO for 20 min. The cells were then stimulated with EGF (50 ng/ml) for the indicated times or left untreated. The cytosolic extracts were subjected to Western blotting with αpS256FoxO1, αGenFoxO1, or αAKT Abs. (**B**) CoIP of AKT and FoxO1 with or without Peptide #3. HeLa cells were serum starved (0.1% FBS, 18hrs) and then treated with Peptide #3, or its scrambled control, for 2.5 hr. Cells were then either left untreated or stimulated with EGF (50 ng/ml) for 10 min. The cytosolic extracts were subjected to CoIP with αAKT Abs. The amount of interacting FoxO1 was determined by Western blotting with αGenFoxO1 Ab (first panel). The same membrane was blotted with αAKT Ab (second panel). The amounts of FoxO1 in the extracts was determined by Western Blotting (lower panel). Quantification of all experiments was done using densitometric analysis by ImageJ. The average normalized intensity is displayed, error bars represent standard error. $P<0.05$ according to students T test. (**C**) Foxo1 is present in the cytosol in resting cells. HeLa cells were serum starved (0.1% FBS, 18hrs), and then stimulated with EGF (50 ng/ml) for the indicated times or left untreated. Immunofluorescence fixed cells was done with αGenFoxO1 Ab. The nuclei were detected using DAPI, and slides were visualized using a fluorescent microscope (×40 magnification).

Figure 1A

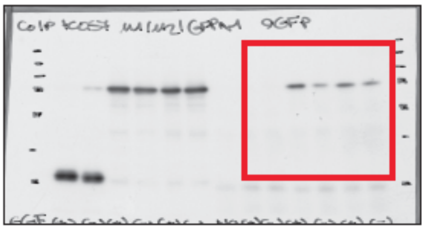

Figure 1B

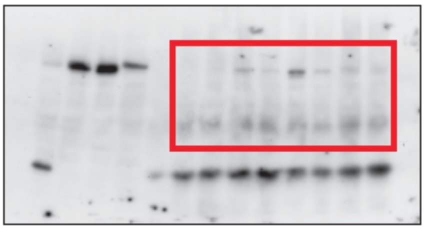

Figure 1C

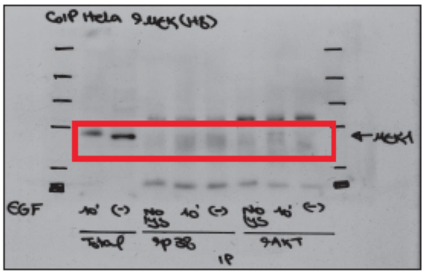

Figure 1D

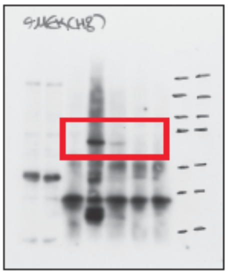

Figure 2C

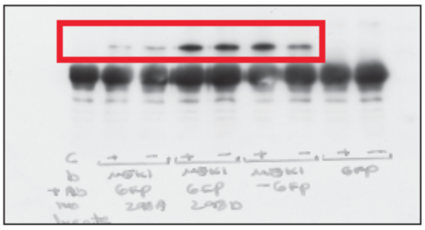

Figure 2F

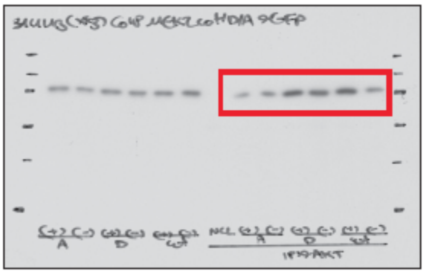

Figure 3A

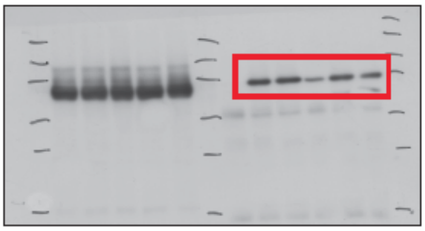

Figure 3D

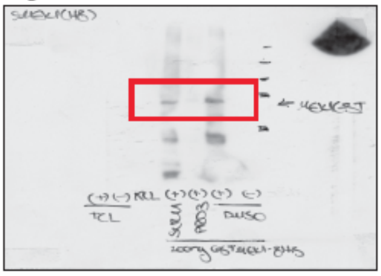

Figure 5B

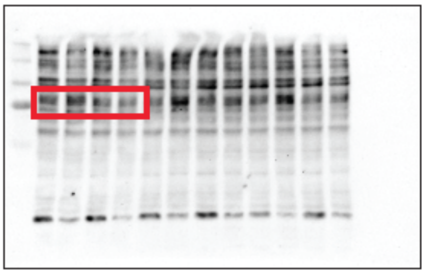

Figure 5D

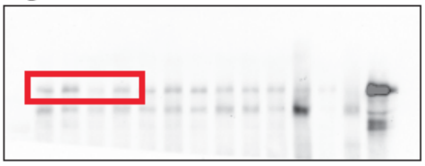

Fig. S8. Uncropped figures
